# Urologic prosthetics: an imaging review of short- and long-term complications

**DOI:** 10.1007/s00261-024-04491-6

**Published:** 2024-07-10

**Authors:** Jared Raikin, Mary Woodruff, Gabriel Meshekow, Nicole D. Debski, Pauline Germaine, Ronak Gor

**Affiliations:** 1 Department of Surgery, Cooper University Healthcare, Camden, NJ USA; 2 Department of Diagnostic Radiology, Cooper University Healthcare, Camden, NJ USA; 3 Cooper Medical School of Rowan University, Camden, NJ USA

**Keywords:** Review, Prosthetics, Genitourinary, Complications, Urology

## Abstract

**Purpose:**

Urologic prosthetics offer significant quality of life enhancements for patients with stress urinary incontinence and erectile dysfunction. Artificial urinary sphincter and penile prosthesis are the most commonly used prosthetics for these patients. Radiographic imaging offers important insight, guiding treatment when patients present with complications. Herein, we pictorialize normal radiographic findings and complications alike.

**Methods:**

We reviewed our IRB-approved prosthetics database, highlighting patients with prosthetic complications with available imaging. We collected imaging from patients without complications for baseline reference.

**Results:**

The radiographic appearance of orthotopic genitourinary prosthetics and a review of short- and long-term complications including hematoma, infection, malpositioning, leak and erosion are pictorialized.

**Conclusion:**

Radiologic imaging serves as a vital complement to history and physical examination, aiding in the identification of complications and potentially streamlining surgical preparations. It is important for radiologists to familiarize themselves with standard prosthetic nomenclature, normal positioning and appearance, along with imaging findings of common complications.

**Graphical abstract:**

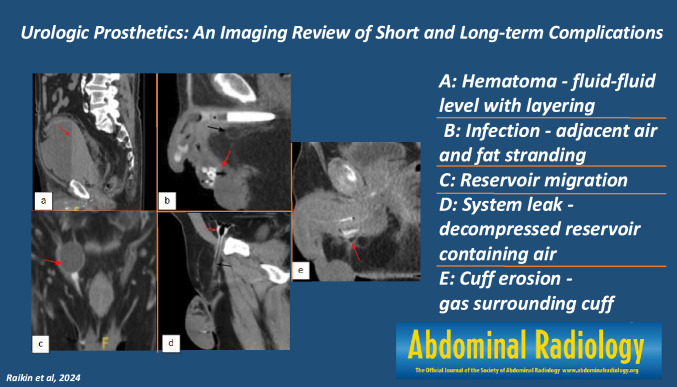

## Introduction

Since the mid-1970s, genitourinary (GU) prosthetics, namely, artificial urinary sphincter (AUS) and penile prosthesis (PP), have served as treatment options for patients suffering from stress urinary incontinence (SUI) and erectile dysfunction (ED) [1]. Every year, approximately 11,500 AUS surgeries [2] and between 15,000 and 20,000 PP surgeries [3, 4] are carried out globally.

Stress urinary incontinence (SUI) is defined as bothersome involuntary, sudden leakage of urine secondary to increased intraabdominal pressure [5]. In men, this condition most commonly results following radical prostatectomy (RP) for prostate cancer [6]. Although evolving with technical refinements, 5–10% of men experience persistent symptoms after one year that prompt them to seek treatment [7]. Conservative management options for SUI include pelvic floor therapy, protective pads, penile clamps, and catheters, while patients with refractory symptoms may undergo surgical evaluation. Generally, patients with persistent moderate to severe SUI are considered candidates for AUS [8].

Erectile dysfunction is defined as the inability to achieve or maintain an erection satisfactory for sexual intercourse, which impacts around 45% of men between 40 and 70 years old [9]. There are many conditions and interventions that contribute to ED, including diabetes, obesity, cardiovascular disease, sleep apnea, spinal cord injury, pelvic radiation, and pelvic surgery. RP can significantly impair erectile function, with some estimating that up to 85% of men develop increasing or de novo ED following RP [10]. Numerous treatment options are available, depending on ED severity, etiology, and patient desires. Lifestyle adjustments and oral phosphodiesterase-5 (PDE-5) inhibitors are common initial therapies. Alternative non-surgical treatments include external vacuum devices, penile injections with vasodilating agents, and intraurethral prostaglandin E1 suppositories. Though not required, most patients who proceed with PP have tried one or more non-surgical approaches before progressing to surgery. When performed by an experienced surgeon, PP offers favorable outcomes and high rates of patient satisfaction [10].

Regarding surgical complications, AUS and PP complications are broadly categorized as infectious or mechanical. These are “hardware” laden procedures with tubing, connections, pumps, valves, and fluid transfer is subject to mechanical strain and failure. Additionally, all prosthetic surgeries include foreign bodies and impose an inherent risk of infection. Given the surgical sites involve the perineum and genitalia, regions with higher bacterial concentration, there is a greater theoretical risk of infection. Magnetic resonance imaging (MRI) offers important anatomic detail with functional PP issues; however, due to access, speed, and often need for MRI compatibility documentation, it is not readily utilized when complications present. Computed tomography (CT) is readily available and rapidly performed, lending itself as the diagnostic modality of choice when complications present [11]. CT provides valuable information that may impact management when combined with history and physical examination. Understanding normal, orthotopic positioning of prosthetic components is important when evaluating complications. Component location and positioning, reservoir volume, presence of abnormal peri-prosthetic fluid, and presence of air and/or fluid around components are all common and important findings radiologists should evaluate [12, 13]. Herein, we outline imaging findings from patients with PP and AUS in normal positions and with various complications.

## Methods

We reviewed our institutional IRB-approved prosthetic urology database, identifying patients receiving AUS and PP. Charts were queried for available CT imaging and clinical notes were reviewed to identify patients with complications. The patient's medical history and physical examination findings, along with imaging results and surgical information, were integrated to develop clinical vignettes accompanied by relevant images for illustrative purposes.

## Results

### Normal AUS positioning and CT

**Fig. 1:**
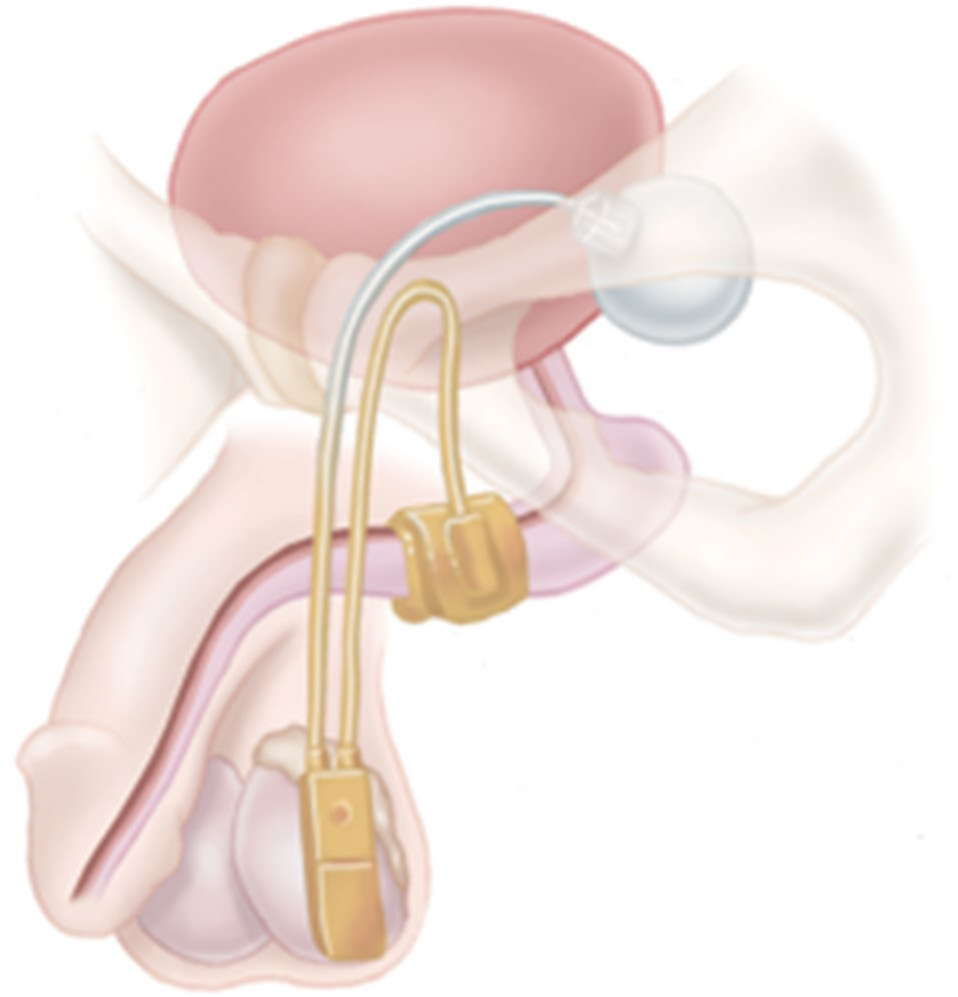
Image of artificial urinary sphincter AMS 800 includes infrapubic PRB, urethral cuff, and scrotal pump [15]. Image adapted from Boston Scientific

**Fig. 2:**
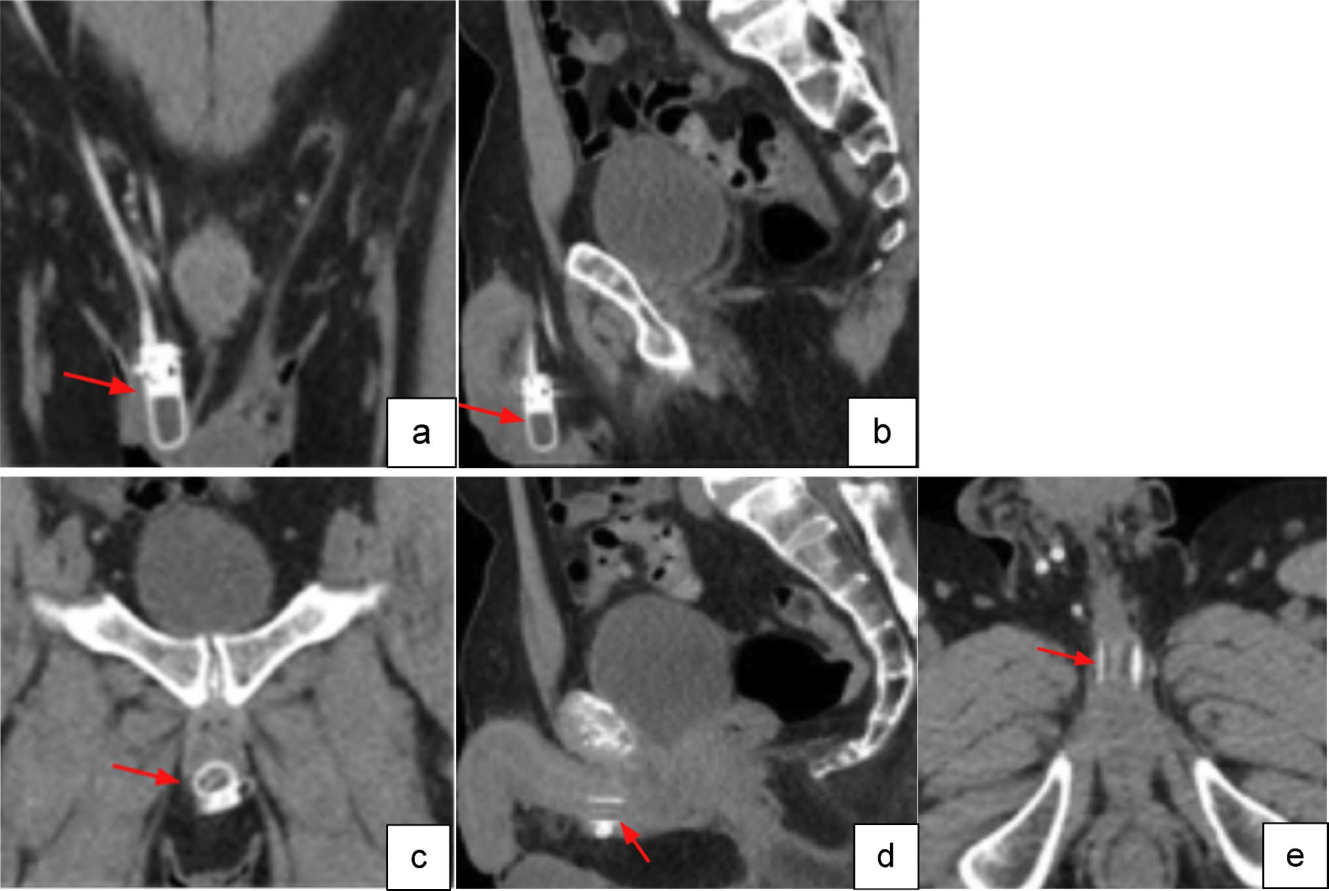
Coronal (**a**), and sagittal (**b**) non-contrast CT images of normal AUS pump location in the lateral scrotum (red arrow). Coronal (**c**), sagittal (**d**) and axial (**e**) non-contrast CT images of normal urethral cuff around the bulbar urethra (red arrows)

Components of the AUS system include an inflatable mid-bulbar urethral cuff, a lateral scrotal pump, a submuscular or retropubic pressure regulating balloon (PRB), and a series of tubing, connecting the pieces (Figs. 1, 2). The system is active at rest with an inflatable fluid-filled cuff surrounding the urethra, which provides continence by circumferentially compressing the urethra. Squeezing the inferior portion of the pump pushes fluid from the pump into the PRB, after which negative pressure from the compressed pump will draw fluid from the cuff into the pump, thereby opening the cuff for urine passage. The cuff will passively fill again over 60–90 s, re-compressing the urethra and providing continence. PRBs are available in 51–60, 61–70, and 71–80 cm H2O pressure, with the 61–70 cm PRB used in most cases. PRBs are typically filled with 23–25 mL sterile saline or diluted contrast [14].

### Normal PP positioning and CT

**Fig. 3:**
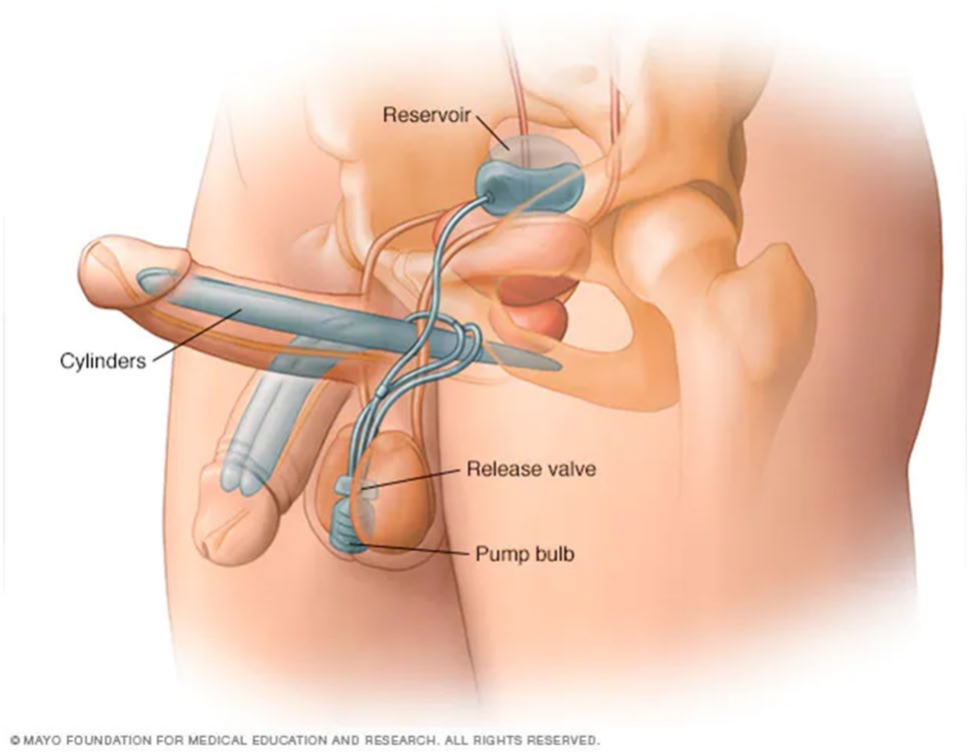
Image of a 3-piece inflatable penile prosthesis with a reservoir, 2 cylinders, and a pump [19]. Image adapted from Mayo Clinic

**Fig. 4:**
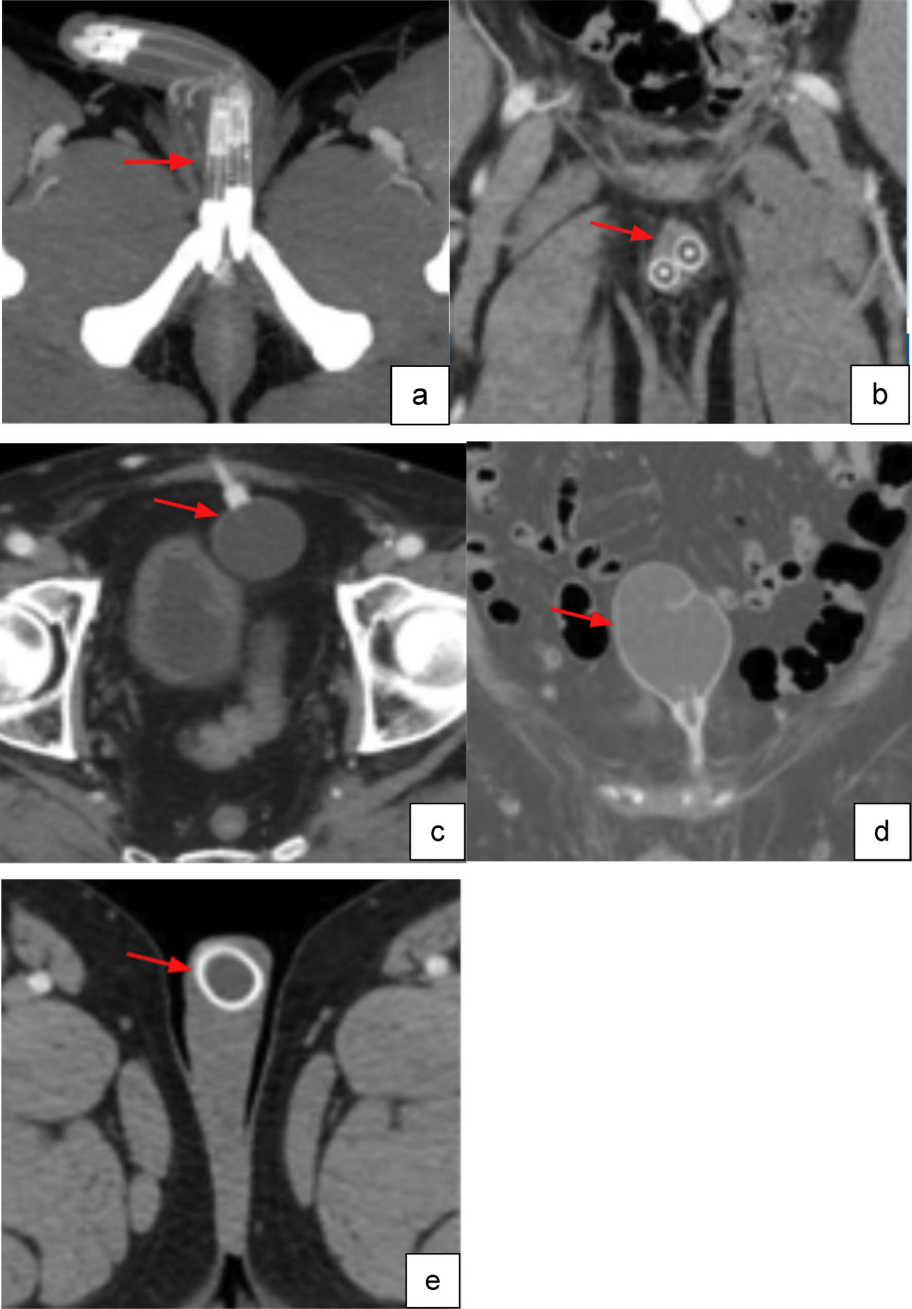
Axial (**a**) and coronal (**b**) CT images with IV contrast of normal three-piece PP paired inflatable cylinders in corpora cavernosa (red arrows). Axial (**c**) and coronal (**d**) CT images of normal PP abdominal placement of reservoir (red arrows). Axial (**e**) CT image of normal PP pump location in the midline inferior scrotum (red arrow)

Penile prostheses are available as inflatable and semi-rigid devices. Semi-rigid devices are malleable cylinders that are inserted into the penile corpora cavernosa with no other components or connections. The corpora cavernosa are paired cylindrical structures in the penis that are joined by a communicating septum in the penis and separate as they course posteriorly in the perineum as crural bodies, inserting to the ischiopubic rami [16]. Inflatable devices are available as two-piece and three-piece devices; most patients in the United States receive three-piece devices featured in this study. Three-piece PP contains custom-sized, paired cylinders within the penile corpora cavernosa, a pump located in the scrotum, and a saline-filled reservoir in the retropubic space (space of Retzius), or under the rectus abdominus or external oblique musculature (Figs. 3, 4). Most PP in the United States are produced by Coloplast and Boston Scientific. Coloplast PP are comprised of Bioflex®, a proprietary material along with silicone and polyurethane. These cylinders are hydrophilic, allowing absorption of custom antibiotic solutions the implant is soaked in prior to insertion. Boston Scientific PP are comprised of three-layer cylinders sourced from silicone and fabric along with a parylene coating. Boston Scientific three-piece implants are non-hydrophilic but are available with an Inhibizone™ antibiotic impregnated silicone comprised of rifampin and minocycline. All three-piece PP pumps are designed with three exit tubes, one allowing fluid transfer between the pump and the reservoir, with the other two allowing fluid transfer between the pump and the corporal cylinders. Reservoirs are custom-filled and available in various shapes, typically between 60 and 120 mL of saline [17, 18]. When an erection is desired, the pump is squeezed, transferring its fluid into the paired corporal cylinders. Negative pressure from the collapsed pump draws fluid from the reservoir, and the pump is squeezed again. This cycle continues, typically 8–20 times, depending on the cylinder length and manual dexterity, until the patient chooses to stop, or the cylinders reach maximal pressure, precluding further fluid transfer. Various manufacturer-specific lockout mechanisms prevent unintentional fluid transfer that could cause loss of erection or auto-inflation. The pump contains a deflate mechanism that restores fluid flow, allowing transfer from the cylinders to the pump and reservoir. These devices have been implanted since the 1970s and have undergone several design advancements, however, the overall design and function of these devices have remained constant for several decades.

### PP and AUS complications and CT findings

#### Short term

##### Postoperative hematoma

Postoperative hematomas are defined by the presence of solid clotted blood within tissue. They are most likely to develop in the readily expandable scrotum. Though only reported to occur in 0.2–3.6% of cases, hematomas are frustrating for patients, and increase the risk of post operative infection [20]. Factors that increase the risk of hematoma include inadequate hemostasis, nonvascular causes of erectile dysfunction, and cases requiring revision [21]. Hematomas typically manifest within the first few weeks after surgery, presenting with pain, swelling, tenderness, and significant ecchymosis. Scrotal hematomas can progress rapidly due to the scrotum’s high compliance and inability to tamponade the area effectively. Small hematomas are often self-limiting and can be managed conservatively with measures such as bedrest, elevating the scrotum, compression dressing, and administering antibiotics [11]. However, large hematomas may require evacuation and control of bleeding.

Though hematomas are apparent on physical exam, CT imaging is often crucial in determining the need for surgical intervention in cases where the diagnosis is less apparent. Patients may develop significant scrotal swelling due to interstitial edema, which is managed conservatively. Imaging provides valuable information regarding the hematoma’s size, location, and composition. CT appearance of hematoma may be variable and depends on acuity. Acute hematoma presents as a hyperdense fluid collection. Additionally, CT can identify foci of active arterial or venous bleeding and the presence of gas and fat stranding, concerning for superimposed infection.

**Fig. 5:**
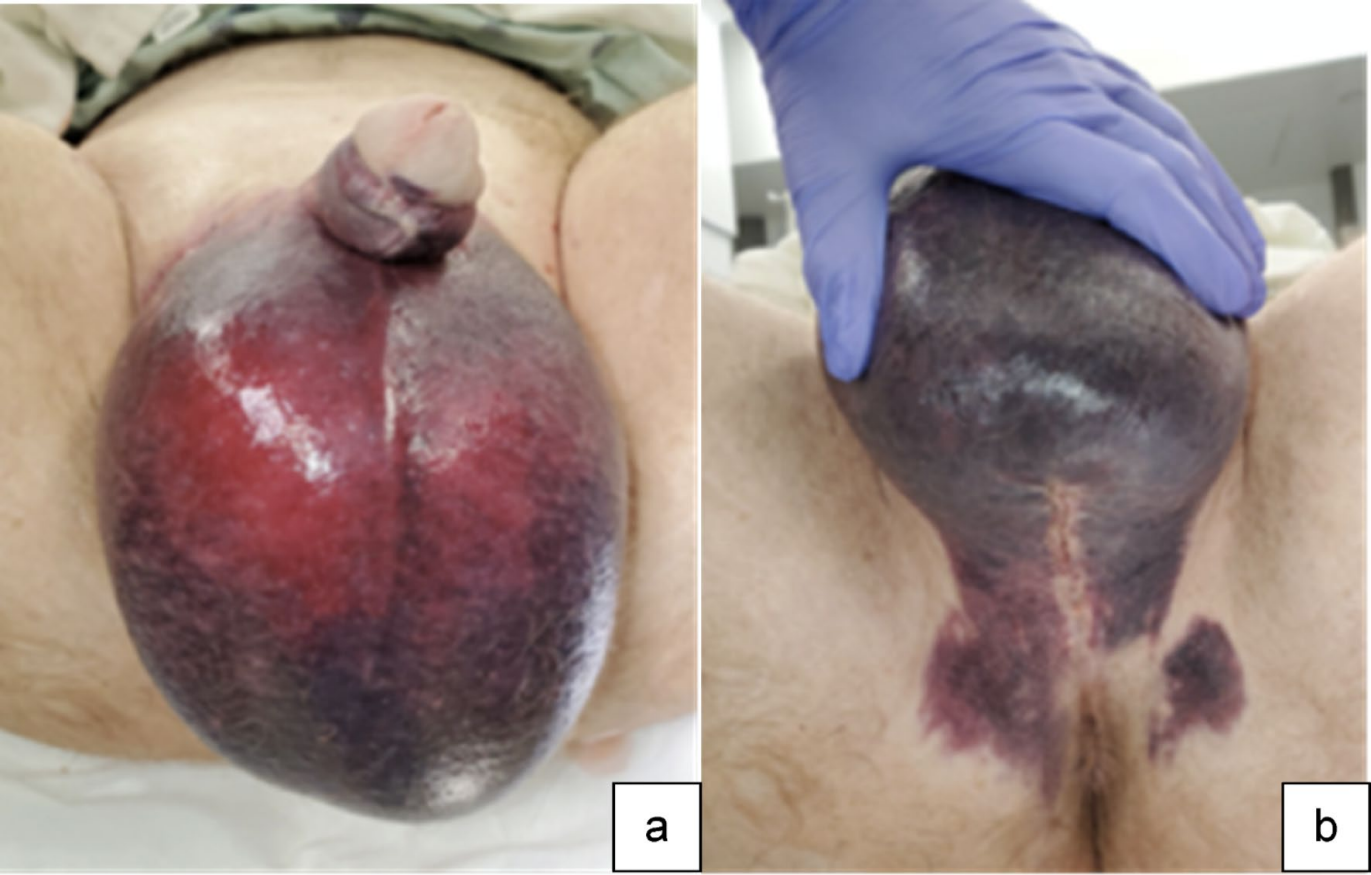
Clinical photo demonstrating scrotal (**a**) and perineal (**b**) enlargement and skin discoloration due to postoperative scrotal hematoma

Case 1: A 61-year-old male presented with penile and scrotal ecchymosis two days after AUS placement (Fig. 5). This patient’s scrotal enlargement, discoloration, and lack of scrotal rugae prompted surgical intervention without imaging, as it was indicated based on physical examination alone.

Teaching point: Scrotal swelling with ecchymosis after prosthetic implantation with disappearance of scrotal rugae indicate need for surgical exploration without delay for imaging.

**Fig. 6:**
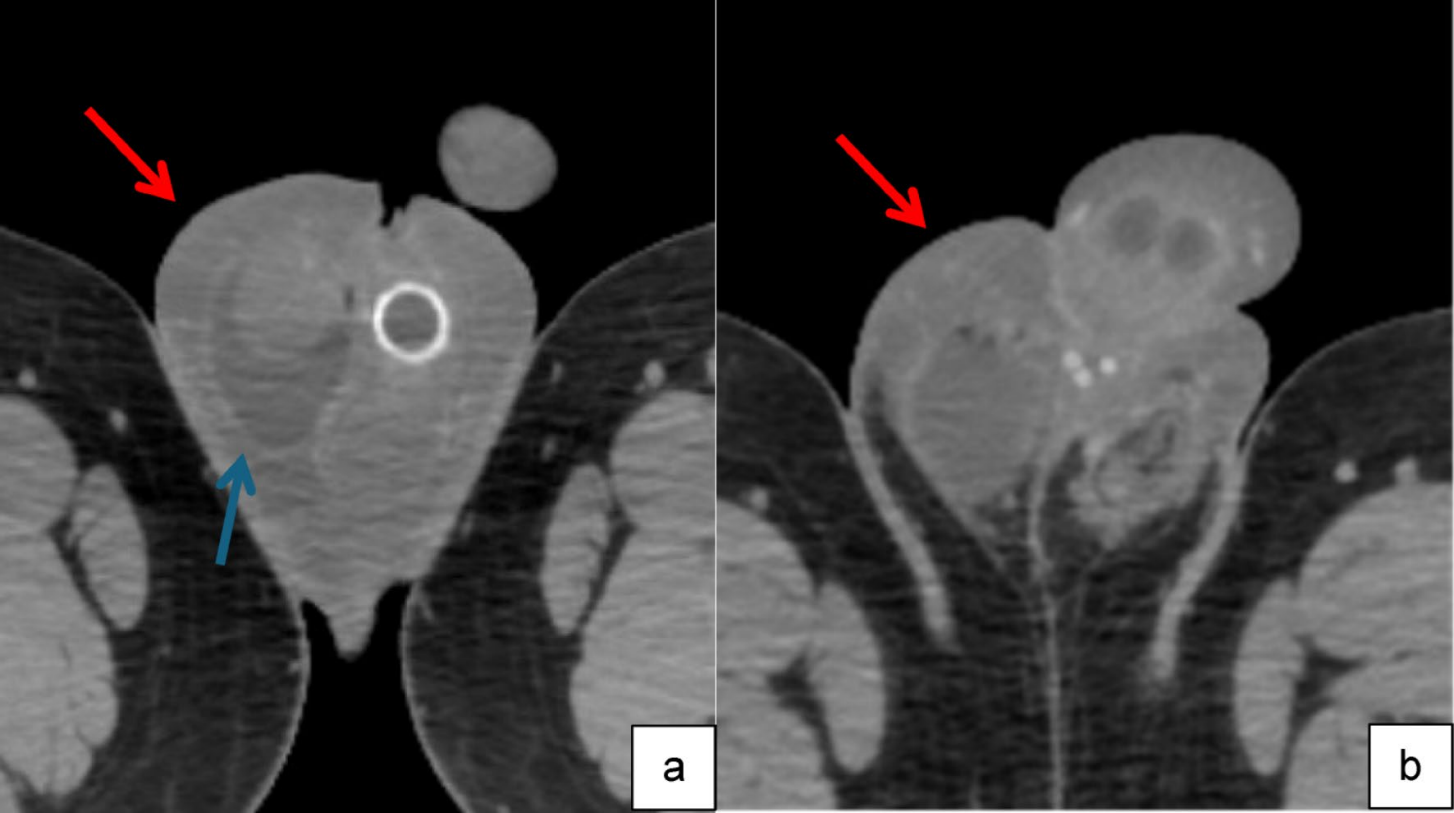
Axial images inferior (**a**) and superior (**b**) from CT pelvis with IV contrast show diffuse penoscrotal skin thickening and subcutaneous edema (red arrow) without discrete hematoma or rim-enhancing fluid collection. Small right hydrocele is incidentally noted (blue arrow). Portion of a PP pump is visible

Case 2: A 60-year-old male underwent uncomplicated insertion of a three-piece IPP. Following the surgery, he experienced moderate bleeding from his scrotal drain, which gradually subsided overnight. On post-operative days 2 and 3, there was an increase in peno-scrotal bruising extending onto the abdominal wall, along with significant swelling. Concern arose regarding whether this was due to hematoma or non-drainable edema. A CT scan of the pelvis with IV contrast revealed diffuse peno-scrotal edema without a discrete hematoma (Fig. 6). Consequently, this patient was treated conservatively, leading to complete resolution of swelling, and bruising one-month following surgery.

Teaching point: Imaging can differentiate post-operative edema from drainable hematoma leading to conservative treatment as opposed to surgical exploration.

**Fig. 7:**
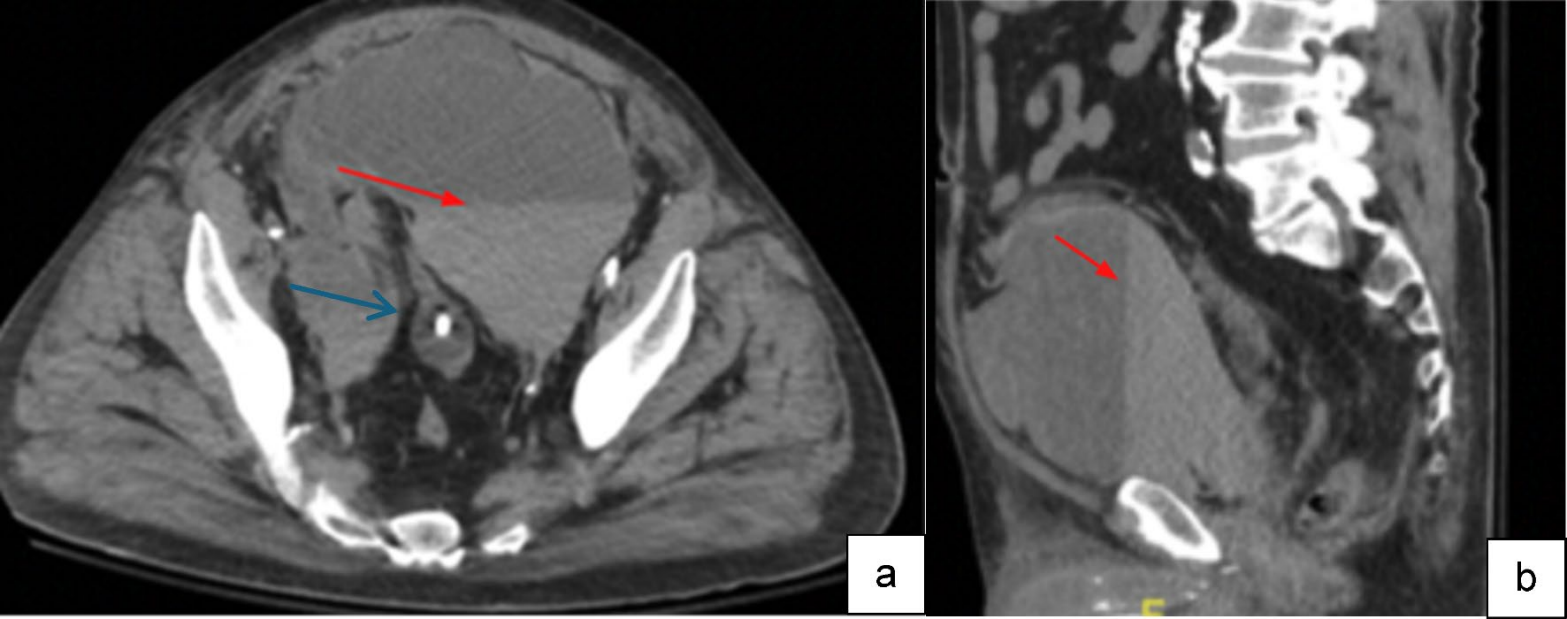
Axial (**a**) and sagittal (**b**) non-contrast CT images, showing large fluid collection in the pelvis with fluid–fluid level, representing layering hematocrit level (red arrows), and compatible with postoperative hematoma. This collection produced significant regional mass effect, displacing the urinary bladder, decompressed around the foley catheter, posterior in the pelvis (blue arrow)

Case 3: A 79-year-old male on warfarin presented with suprapubic pain one week after undergoing AUS placement. Initial physical exam revealed lower abdominal and flank tenderness. CT imaging revealed the presence of a large retropubic collection, with a fluid–fluid level (Fig. 7). Large hematomas in the space of Retzius create a retroperitoneal compartment syndrome that may result in ureteral and/or pelvic vasculature obstruction. This patient developed oliguria with bilateral hydronephrosis. Physical examination alone was insufficient to proceed with operative intervention. CT imaging facilitated diagnosis and guided the decision to proceed with surgical exploration and hematoma evacuation in this case.

Teaching point: Pelvic fluid collection after prosthetic implantation with dependent layering is consistent with hematoma and often requires surgical intervention.

##### Postoperative infection

Postoperative infections involving urologic prosthetics are estimated to occur in 0.46–5.3% of cases [25]. Risk of infection is higher in patients undergoing revision surgery, with poorly controlled diabetes, or spinal cord injury [22]. Preoperative optimized blood sugar control is important in decreasing the risk of skin breakdown and infection [23]. While uncommon, infections are a feared complication due to their potential to necessitate explantation of the prosthesis, as well as lead to further issues such as penile shortening and cavernosal fibrosis [11]. Patients with infections can present with localized swelling, erythema, pain, fever, leukocytosis, purulent drainage, and extrusion of the prosthesis. From the imaging perspective, surgeons are looking for signs of infection, including fat stranding, foci of gas within or adjacent to the prosthesis and any rim-enhancing fluid collections. In the immediate post-operative period, fat stranding may be present due to tissue response from cautery and dissection. However, when present and paired with sub-clinical signs and symptoms of infection, this finding raises the concern for a developing infection. Typically, there should be no soft-tissue materials lining any of the prosthetic components, which may be a subtle sign of infection. These findings impact decision making by necessitating immediate surgical explantation of prosthetics and antibiotic use. In some cases, immediate antibiotic washout of the corpora cavernosa and all surgical spaces with a new device insertion may be considered. This decision is made based on surgeon preference and patient characteristics.

**Fig. 8:**
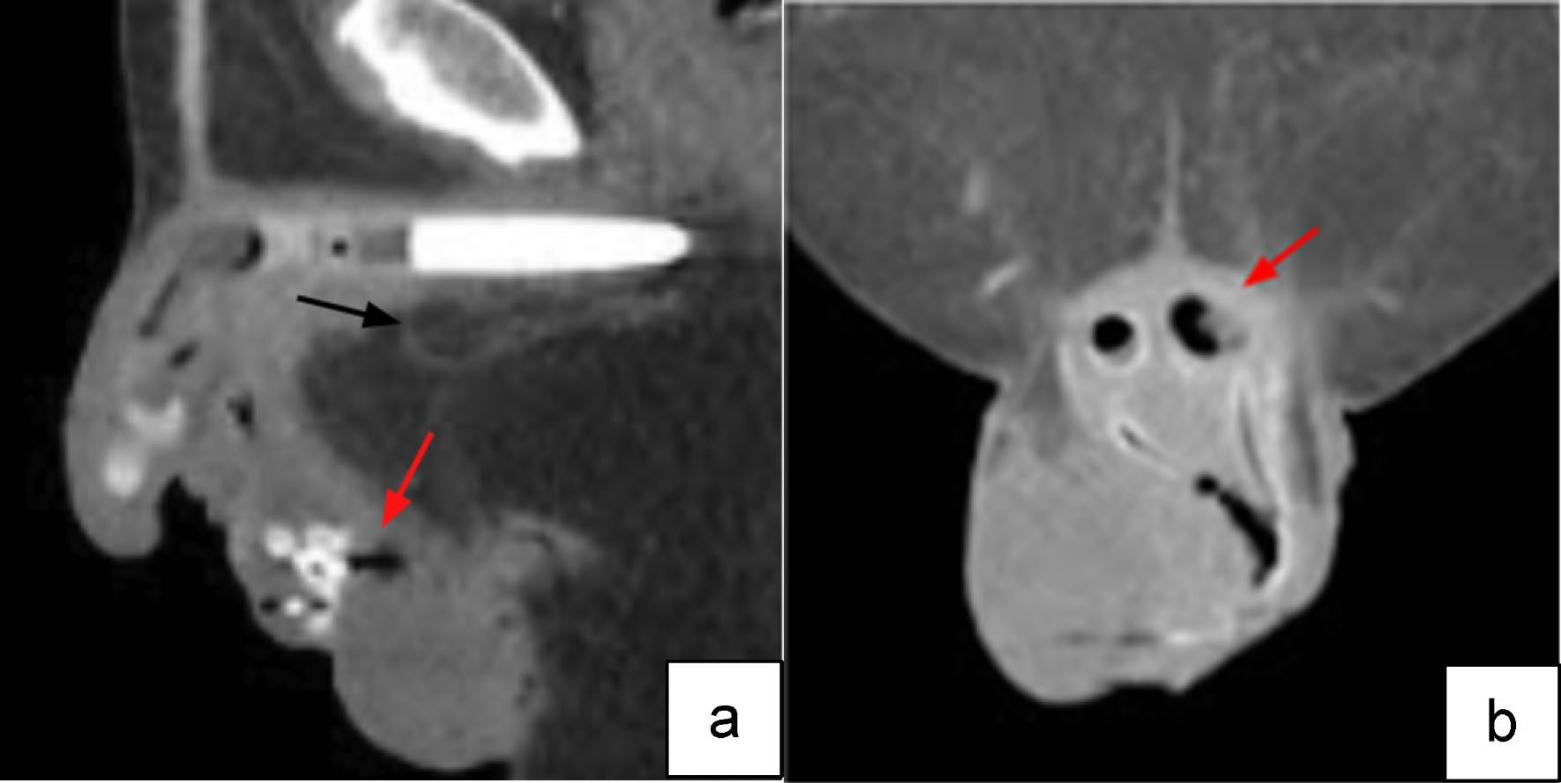
Sagittal (**a**) and coronal (**b**) CT images demonstrate gas (red arrows) within the scrotum and corpora cavernosa components of the PP with surrounding fat stranding (black arrows) compatible with infection

Case 4: A 72-year-old male on warfarin presented with penile pain approximately 3 weeks after PP placement. CT imaging was obtained due to concern of prosthetic infection and demonstrated gas and fat stranding around the prosthesis with foci of gas within the components of the PP (Fig. 8). The presence of gas around the device on imaging in the postoperative period raised concern for infection requiring immediate surgical explantation.

Teaching point: Though air around the components may be seen in the immediate post-operative period, the presence of significant air, gas and fat stranding adjacent to prosthetics in the short and intermediate post-operative periods, when combined with equivocal physical exam findings raises concern for infection and need for prompt surgical explanation.

**Fig. 9:**
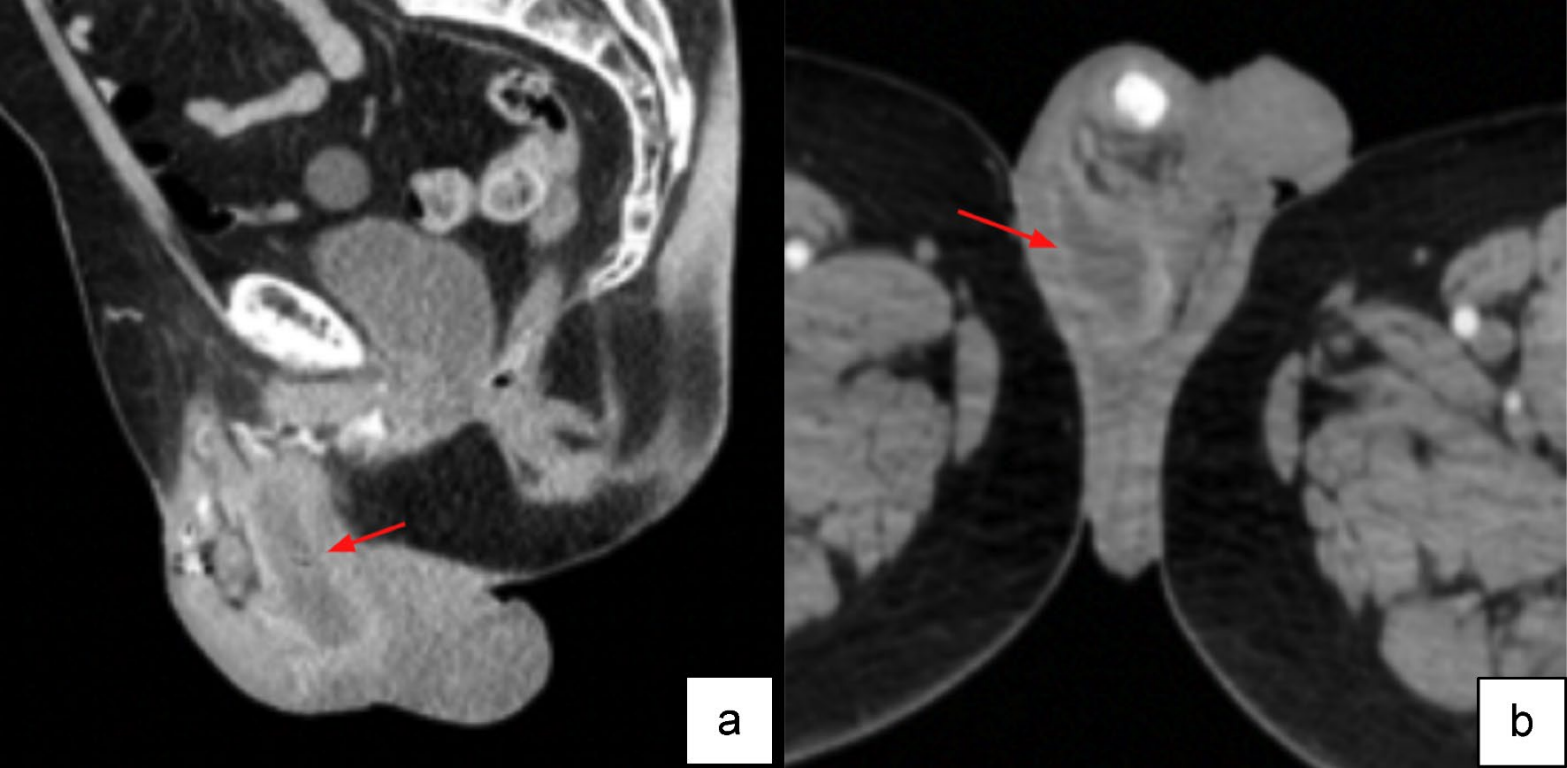
Sagittal (**a**) and axial (**b**) CT images with IV contrast show an intrascrotal peripherally enhancing fluid collection (red arrows), consistent with an abscess

Case 5: A 74-year-old male presented one month after undergoing AUS revision surgery with malodorous drainage from the right hemiscrotum. Patient was also found to have leukocytosis, raising concern for an underlying infection. Imaging was ordered to identify extent of infectious involvement, demonstrate any fluid collection and define presence of prosthetic involvement. CT demonstrated a scrotal rim-enhancing collection adjacent to the IPP, consistent with a scrotal abscess (Fig. 9), requiring an immediate surgical removal of IPP. This emphasizes the importance of correlating clinical signs and symptoms such as fever and leukocytosis with imaging findings. Administration of intravenous contrast material for CT imaging helps in visualization of the fluid collections in this relatively small and highly compartmentalized region.

Teaching point: Clinical and laboratory findings help guide decision management. Administration of intravenous contrast material for CT helps in visualization of postoperative fluid collections.

**Fig. 10:**
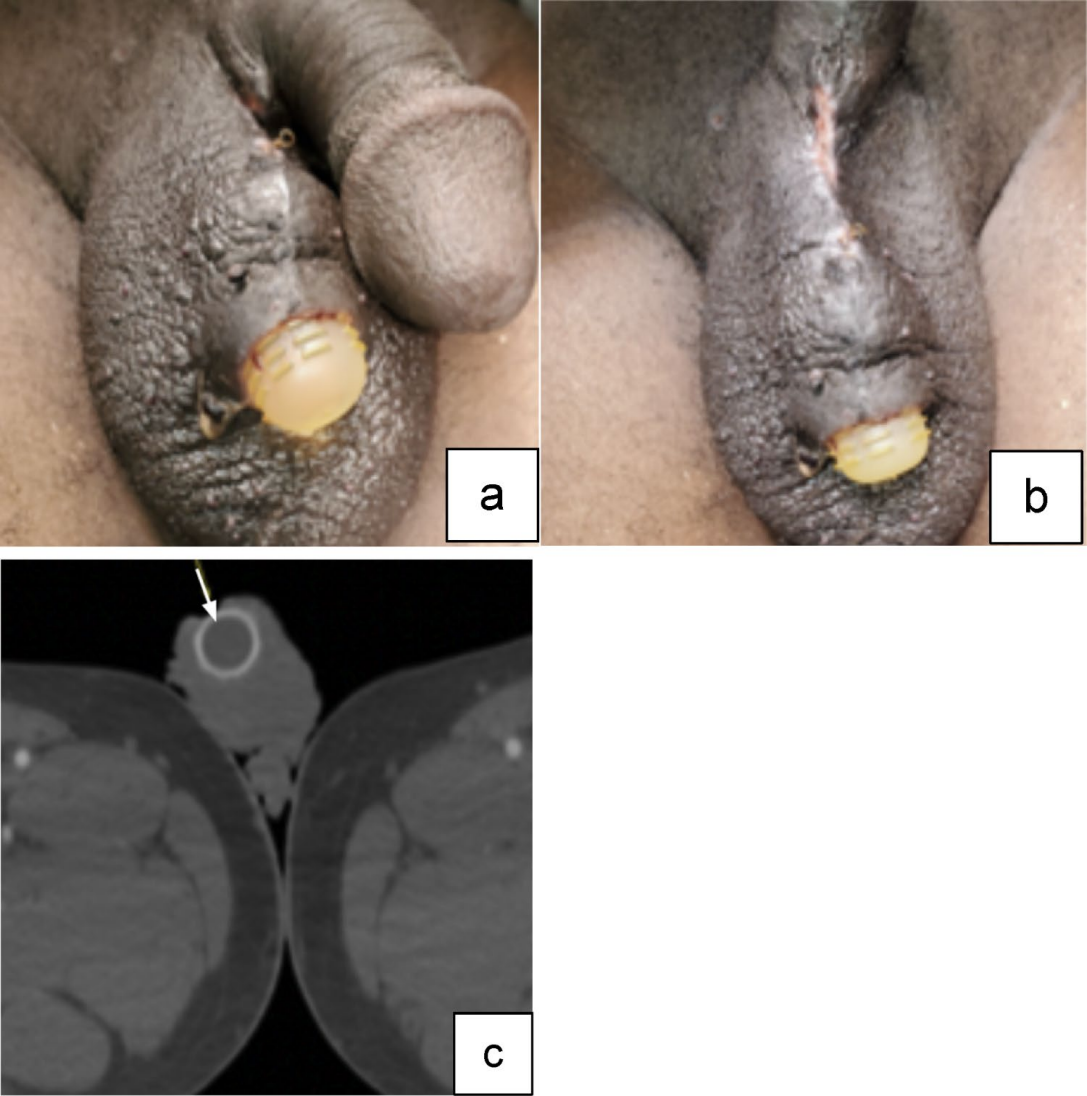
Photos show skin breakdown with the penile pump prosthesis protruding through the skin of the scrotum (**a**, **b**). Axial CT image with IV contrast (**c**) shows absence of skin barrier overlying the pump (white arrow)

Case 6: A 56-year-old male underwent PP placement, with development of postoperative abscess that necessitated drainage. He subsequently presented to the office for wound evaluation and was found to have a region of skin breakdown around the scrotal pump on physical exam. Imaging was obtained to assess for deeper involvement, again confirming a lack of skin barrier overlying the pump (Fig. 10). No scrotal or perineal fluid collections were seen on CT. The entire prosthetic device was removed due to the assumption that if any component has extruded from the skin, the entire device is considered contaminated.

Teaching point: Visible components of any prosthetic are an indication for complete IPP removal.

#### Long term

##### Component migration

An uncommon complication that can arise with prosthetic surgery is migration of prosthesis components, such as the reservoir or the pump. Migration complications are rare, found in 1.5% of cases, based on a retrospective study [6]. Reservoir migration can occur when the space created through the inguinal ring is capacious enough that, with increased abdominal pressure, the reservoir completely or incompletely herniates through the external ring [24]. Pump migration can result from inadequate closure of the scrotal space or secondary to other complications, such as hematoma. Though reservoir herniation typically does not interfere with device functionality, it may cause discomfort warranting surgical correction. Patients may present with difficulty using the pump or an inguinal bulge. Imaging can facilitate intraoperative decision making as the exact location of the migrated reservoir in relation to the external ring can impact surgical approach. Additionally, if the reservoir was inserted in a submuscular location, subtle palpability of the reservoir may be normal, especially in patients with a thin abdominal wall. Imaging plays a key role in the surgical planning and management of these cases as it provides information about the exact anatomic location of the migrated device components and any alterations in normal anatomy.

**Fig. 11:**
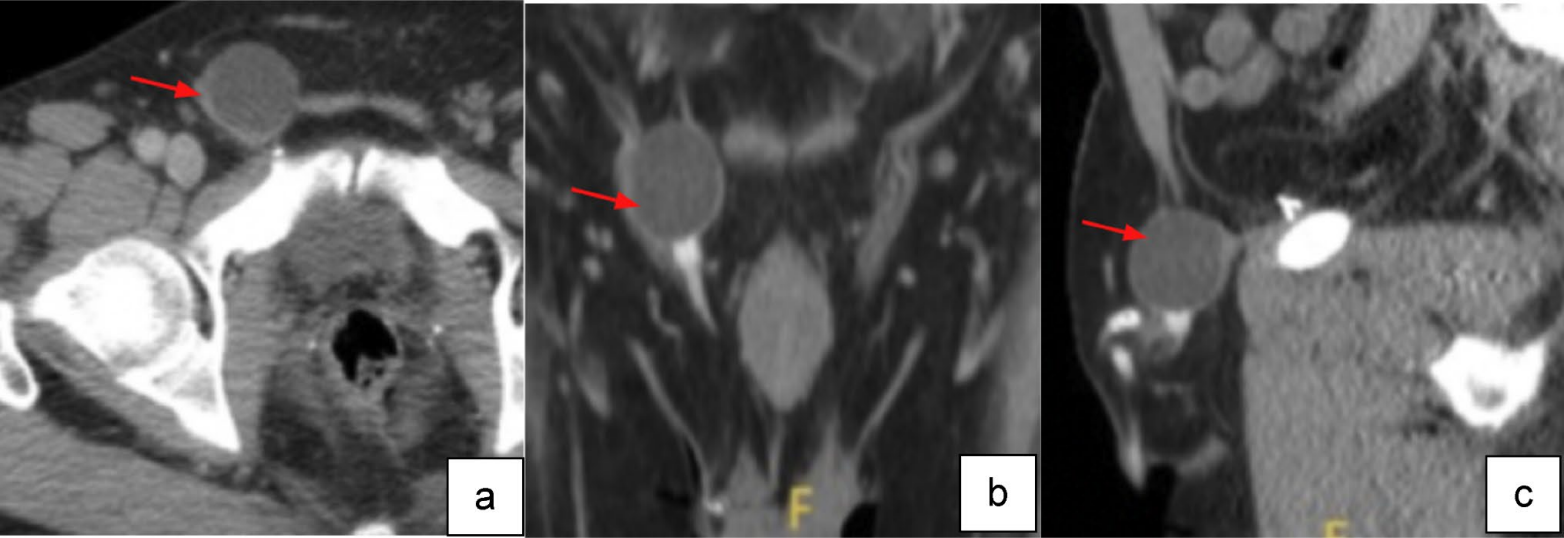
Axial (**a**), coronal (**b**) and sagittal (**c**) CT images with IV contrast show reservoir of IPP in the inguinal canal (red arrows)

Case 7: A 57-year-old male with a painless inguinal mass presents 9 months after PP placement (Fig. 11). CT imaging was obtained due to inconclusive physical exam and demonstrated the reservoir herniation into the inguinal canal. Surgical intervention was performed to reposition the reservoir to avoid device erosion into adjacent structures.

Teaching point: Detailed imaging descriptions of prosthetic component positioning can aid in identifying malpositioning and determine need for surgical intervention.

**Fig. 12:**
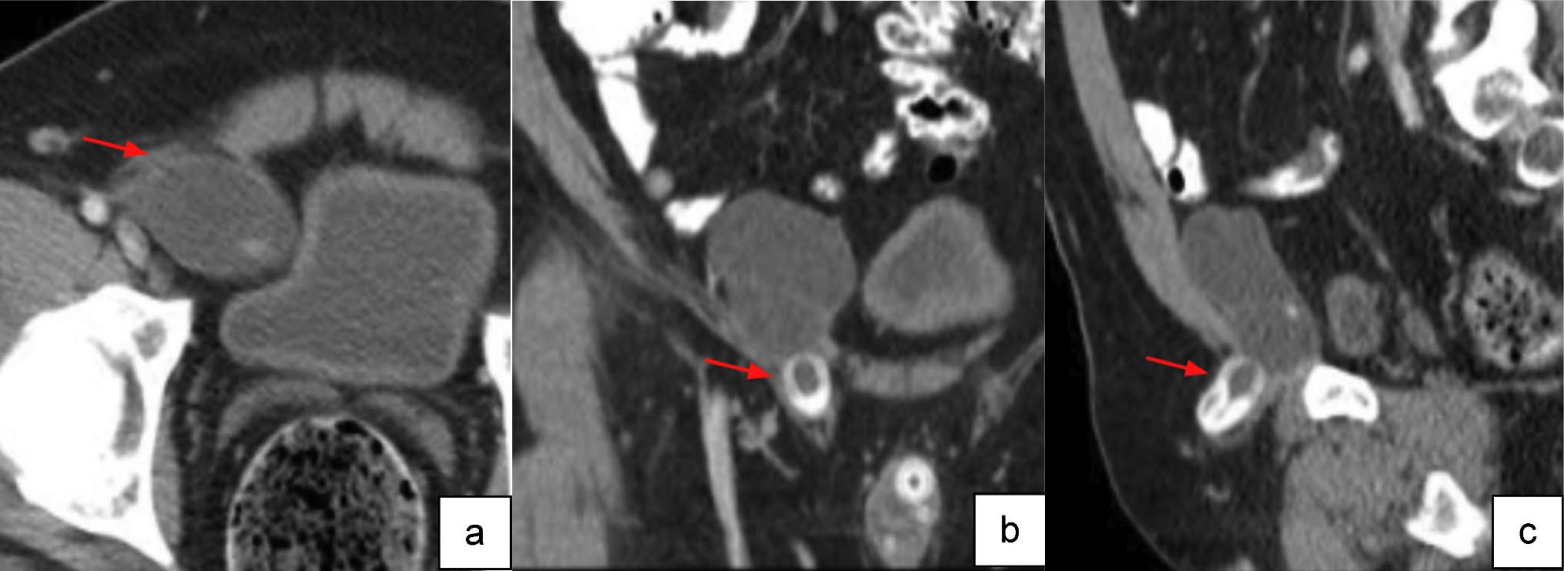
Axial (**a**), coronal (**b**) and sagittal (**c**) CT images with IV contrast show IPP reservoir partially extending into the inguinal canal (red arrows)

Case 8: A 69-year-old male was incidentally noted to have a herniation of the IPP reservoir, partially extending into the inguinal canal (Fig. 12). The patient was asymptomatic with minimal device herniation and no surgical intervention was required.

Teaching point: Incidental findings of prosthetic reservoir herniation without adjacent tissue damage in an asymptomatic patient do not require intervention.

##### System leak

A system leak is due to a mechanical failure or tear in a cylinder or tube of the prosthesis, leading to fluid leakage from the system. Time since implantation is the biggest risk factor, with mechanical survival, or lack of failure, decreasing over time with rates for PPs at 97.6%, 86.2%–93.2%, 68.5%–85.0%, and 59.7%–79.2% at 3, 5, 10, and 15 years, respectively [25]. System leak usually results in device malfunction and is an indication for operative prosthetic removal and replacement. Clinically, patients with a leak from an AUS system may notice increased incontinence, while patients with leaks from PP implants may observe inadequate fluid cycling during inflation. A leak may also be suspected when a low reservoir volume is detected on imaging. The standard amount of fluid is 22-23 cc in AUS PRBs and varies between 80 and 120 cc in PP reservoirs. Gas within or surrounding prosthetic components is present in 53.8% of cases with fluid leak [11]. The presence of intra-component gas on imaging in a long-term device is suggestive of a system leak, as all urologic prosthetics are closed systems and should not contain any gas after insertion.

**Fig. 13:**
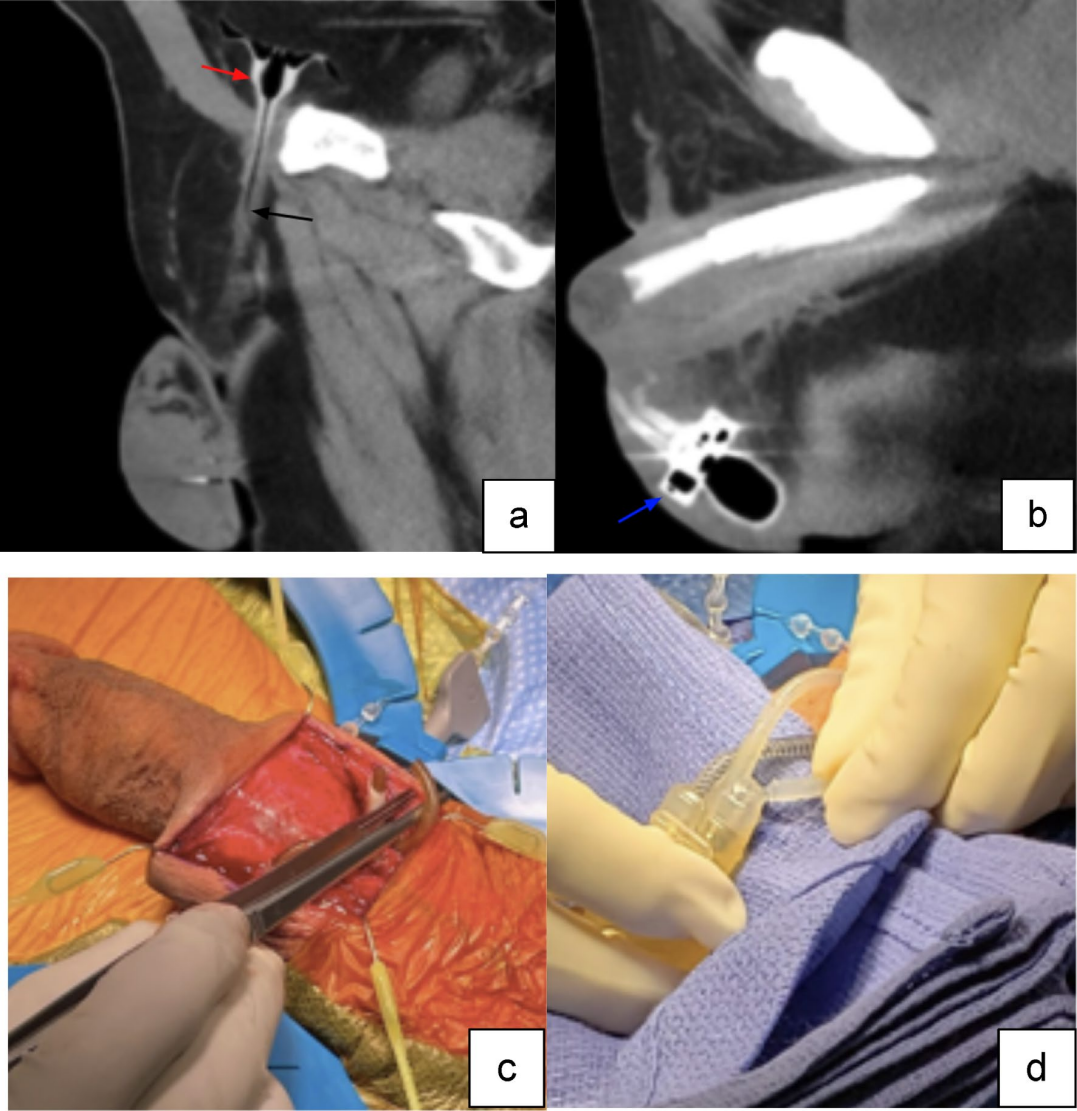
Sagittal (**a**) and axial (**b**) CT images without contrast demonstrate a compressed PP reservoir containing air (red arrow), fractured free-floating tubing (black arrow), and air within the scrotal pump (blue arrow). Intraoperative photos of penoscrotal incision at time of repair demonstrate multiple fractures of prosthetic connection tubing (**c**, **d**)

Case 9: A 67-year-old male presented with an inability to produce an erection eight years after placement of a 3-piece PP. CT imaging was ordered due to concern for device malfunction and revealed a compressed reservoir and gas within the scrotal pump (Fig. 13a, b). CT findings confirmed device malfunction, leading to operative removal and replacement of the prosthetic with intraoperative identification of fractured tubing (Fig. 13c, d).

Teaching point: GU prosthetics are closed systems, any gas within the system is consistent with system leak requiring elective surgical reimplantation.

**Fig. 14:**
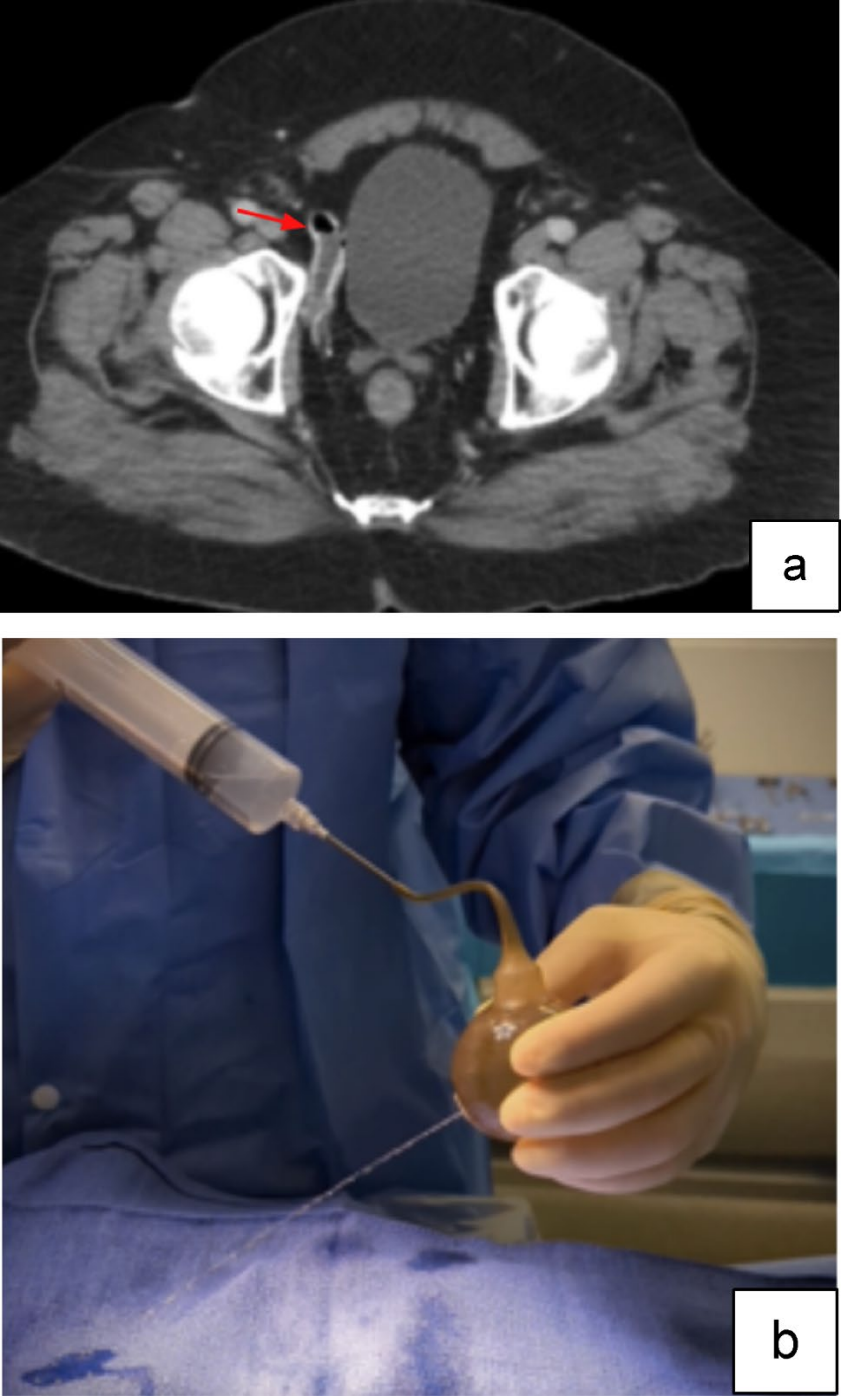
Axial CT image with IV contrast (**a**) shows a collapsed reservoir with an air-fluid level, compatible with system leak (red arrow). Intraoperative photo demonstrates pinpoint fluid leak from reservoir (**b**)

Case 10: A 74-year-old male presented after remote PP placement with an inability to produce an erection. CT, obtained due to concern for device malfunction, revealed a collapsed reservoir with an air-fluid level (Fig. 14a). Imaging findings confirmed system leak and guided surgeon’s decision making in operative removal and replacement, with reservoir leak confirmed during the repair procedure (Fig. 14b).

Teaching point: Volume measurement of fluid in PRB in AUS and reservoirs in PP can assist with diagnosis of system fluid leaks in equivocal cases.

##### Urethral cuff erosion

Artificial urinary sphincter cuff erosions are devastating complications where the circumferential urethral cuff erodes partially or completely into the urethral lumen. This complication is estimated to occur in upwards of 18% of AUS cases [26]. Early erosions are frequently associated with unrecognized injuries during surgery, while later erosions often result from catheter instrumentation while the cuff remains inflated. Risk factors include history of pelvic radiation, hypogonadism, previous treatment for bladder neck contracture or urethral stricture, or prior treatment with a urethral stent [19]. Erosions necessitate surgical explantation, urethral repair, and delayed implantation after a lengthy period of urethral healing and urinary diversion [27]. Patients often present with sudden changes in their continence, with some developing retention of urine while others may note increased incontinence. Cystoscopy is performed, which typically identifies cuff material within the urethral lumen. Urologists use presence of adjacent soft tissue gas, contrast extravasation, and visualization of erosion cystoscopically to determine the need for surgical explantation.

**Fig. 15:**
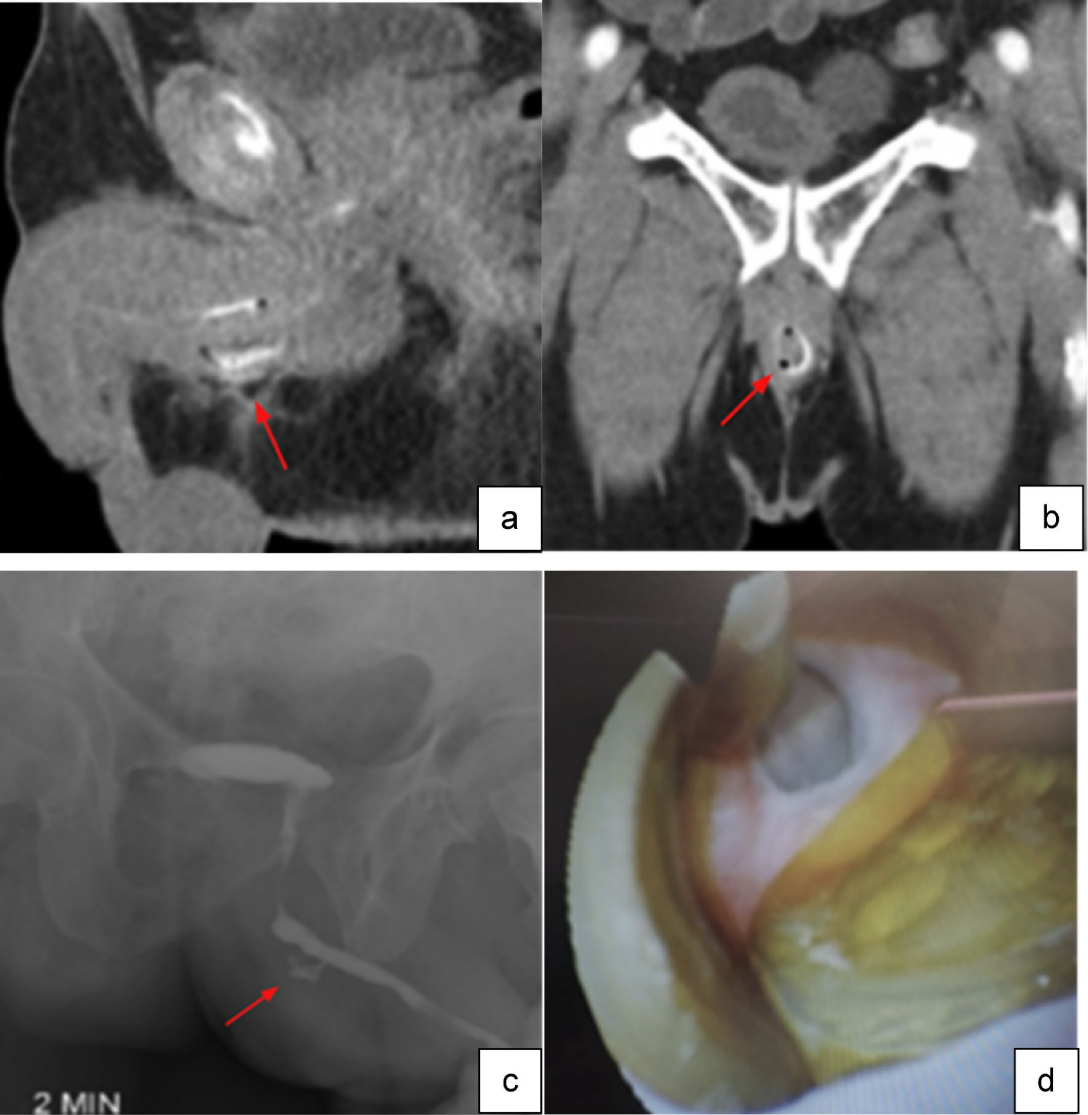
Sagittal (**a**) and coronal (**b**) CT images with IV contrast demonstrate foci of gas and fat stranding at the base of the penis concerning for cuff erosion (red arrows). Portable radiograph after retrograde urethrogram (**c**) shows contrast extravasation at anterior penile urethra in the region of foci of gas on CT (red arrow). Intraoperative cystoscopic view (**d**) of circumferential artificial urinary sphincter erosion

Case 11: A 72-year-old male with AUS placement eight years prior presented with penile pain and incontinence shortly after an unrelated surgery that involved traumatic catheterization in the setting of an inflated cuff. Retrograde urethrogram and cystoscopy were performed due to concern for traumatic urethral erosion. CT scan revealed foci of gas and inflammatory changes surrounding the bulbar urethra (Fig. 15a, b), retrograde urethrogram demonstrated contrast extravasation at the same level (Fig. 15c), and cystoscopy confirmed circumferential AUS erosion (Fig. 15d). To allow the affected tissues to heal, the eroded device was removed and reimplantation considered for a later time.

Teaching point: Foci of gas surrounding the cuff of AUS are concerning for cuff erosion and should prompt further workup including retrograde urethrogram and cystoscopy.

## Conclusion

Genitourinary prosthetics, including PP and AUS, provide life changing care for patients with impotence and incontinence. Complications, including hematoma, infection, component migration, system leak, and cuff erosion are uncommon, but often require prompt surgical intervention. CT imaging plays a crucial role in prompt diagnosis, and it is imperative for radiologists to possess familiarity with normal and abnormal positioning of these devices. Cases presented in this paper, regarding short and long-term complications of prosthetics and their associated radiographic findings, are vital to informing care. With more of these prosthetics being implanted and remaining in place for extended periods, they are likely to become more prevalent in CT imaging studies. Enhanced understanding and recognition of common complications, along with their subtle imaging findings, are essential for facilitating prompt decision-making.
